# The Role of Gut Microbiota in Acute Myeloid Leukemia

**DOI:** 10.3390/jcm15103571

**Published:** 2026-05-07

**Authors:** Lydia Maria Inglezou, Theocharis Konstantinidis, Aikaterini Skeva, Bouse Malkots, Emmanouil Panagiotopoulos, Vasiliki Sakka, Emmanouil Spanoudakis, Maria Panopoulou, Ioannis Kotsianidis, Konstantinos Liapis

**Affiliations:** 1 Department of Hematology, Democritus University of Thrace Medical School, 68 100 Alexandroupolis, Greece; malkotsbouse@gmail.com (B.M.); manolispan44@gmail.com (E.P.); vasilia.sakka@gmail.com (V.S.); espanoud@med.duth.gr (E.S.); ikotsian@med.duth.gr (I.K.); koliapi@med.duth.gr (K.L.); 2 Laboratory of Microbiology, Democritus University of Thrace Medical School, 68 100 Alexandroupolis, Greece; tkonsta@med.duth.gr (T.K.); skevakaterina@hotmail.com (A.S.); mpanopou@med.duth.gr (M.P.)

**Keywords:** acute myeloid leukemia (AML), human intestinal microbiome, gut microbiota, dysbiosis, immune system, diet, allogeneic stem-cell transplantation, infections

## Abstract

Gut microbiota plays a crucial role in regulating immune system function and shaping immunological responses to pathogens capable of causing infections. Alterations in the composition of the intestinal microbiome are associated with immune system dysfunction and increased susceptibility to infections. Patients with acute myeloid leukemia (AML) are highly susceptible to infections due to immune system deregulation caused by the disease itself, as well as chemotherapy-induced bone marrow aplasia. In these patients, gut microbiota dysbiosis and reduced microbial diversity (i.e., imbalances in the composition and function of intestinal microbes) result from multiple factors, including the underlying disease, neutropenia, dietary factors, use of antibiotics, chemotherapy regimens and prolonged hospitalization. Chemotherapy, for instance, induces damage to the intestinal mucosa and disrupts the epithelial barrier, resulting in deregulation of the intestinal microbiome. Previous studies have reported alterations in the human intestinal microbiome in patients with AML undergoing chemotherapy. Of particular interest is the capacity of some commensal bacteria to modulate the tumor microenvironment and response to chemotherapy. Moreover, increased mortality and reduced overall survival have been reported in patients who have undergone allogeneic stem cell transplantation and exhibit decreased gut microbiome diversity at the time of transplantation. These findings indicate that the composition of gut microbiota may play an important role in the prognosis of AML, especially in relation to therapeutic response. This narrative review summarizes new research into the role of the intestinal microbiome and the underlying alterations observed in patients with AML, resulting from the disease and therapeutic interventions and outlines strategies to improve its function and outcomes.

## 1. Introduction

Acute myeloid leukemia (AML) is a clonal hematopoietic malignancy characterized by the infiltration of myeloid blasts in the bone marrow. AML can affect people of all ages; however, it is most common in older individuals and represents the most frequent type of acute leukemia in adults. The prognosis of AML is determined by the patient’s age and genomic profile. The disease is categorized into favorable, intermediate or high-risk groups based on cytogenetic abnormalities and specific gene mutations. Patients with AML are highly vulnerable to infections due to disease-related immune dysfunction and chemotherapy-induced bone marrow aplasia. Consequently, these patients require hospitalization for febrile neutropenia and are treated with broad-spectrum antibiotics [1].

The gut microbiota consists of a large number of diverse microbial species that reside in the distal gastrointestinal tract and plays a significant role in maintaining homeostasis and epithelial barrier integrity [1]. Additionally, it modulates immune system function and immunological responses to pathogens responsible for infections [2]. Alterations in the composition of the intestinal microbiome, referred to as dysbiosis, are associated with immune system dysfunction and increased susceptibility to infections [3].

In recent years, there has been an increase in interest in the interaction between gut microbiota and antineoplastic drugs. Patients with AML generally receive stereotypical treatment: younger patients receive cytarabine for seven days and anthracycline for three days (known as “7 + 3 induction”), resulting in complete remission in approximately 70% of patients. However, this standard induction regimen is characterized by major disruptions in the intestinal mucosa of patients with AML. It is accompanied by gut damage that promotes bacterial translocation and bloodstream infections, usually caused by Gram-negative bacteria [4]. More specifically, it has been reported that anthracyclines increase the mucosal permeability, resulting in infectious complications [5]. Furthermore, bone marrow suppression following intensive chemotherapy results in a decline in the white-cell count, thereby increasing the infection risk. Exposure to multiple antibiotics during the period of neutropenia also contributes to the gastrointestinal epithelial barrier dysfunction [4].

## 2. Composition of Human Intestinal Microbiome Under Normal Circumstances

The human gut microbiota constitutes a highly complex and dynamic ecosystem hosting a vast array of microorganisms that engage in continuous interactions both with each other and with the human host. Its composition is dominated by four major groups (phyla) of bacteria: *Firmicutes*, *Bacteroidetes*, *Actinobacteria* and *Proteobacteria*. The *Firmicutes* to *Bacteroidetes* ratio is widely used as a key indicator of disturbances or imbalance within the gut microbiota [6]. A recent large-scale analysis suggests that the human gut microbiota collectively encompasses more than 35,000 distinct bacterial species. Furthermore, findings from the Human Microbiome Project and the Metagenome of the Human Intestinal tract indicate that the human microbiome may harbor more than 10 million unique, non-redundant genes. Moreover, the phylum *Proteobacteria* includes many potential pathogenic microorganisms, e.g., *E. coli,* which usually occur at very low levels and their scarcity, together with a high relative abundance of *Bacteroides*, *Prevotella* and *Ruminococcus* is considered indicative of a healthy gut microbial community [7]. Gut microbiota dysbiosis results from a disturbance in the equilibrium between commensal and pathogenic microbial populations.

## 3. Human Intestinal Microbiome and Immunity

**Figure 1:**
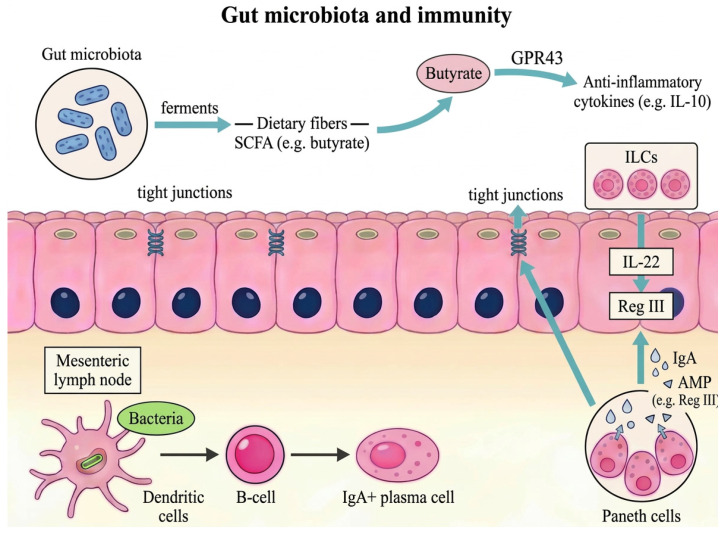
**Interplay between gut microbiota and immunity.** The gut microbiota ferments dietary fibers, leading to the production of metabolites that influence immunity, such as short-chain fatty acids (SCFAs). Butyrate, one of the most important SCFAs, engages GPR43, inhibits the NF-kappa B (NF-κB) signaling pathway and histone deacetylation and promotes the secretion of anti-inflammatory cytokines. Additionally, commensal bacteria enhance tight junctions of the intestinal epithelium, creating a stable structure. IgA and antimicrobial peptides (AMP) such as RegIII, α-defensins and lysozyme secreted by Paneth cells located at the base of the crypts of Lieberkühn in the small intestine, further sustain the integrity of the mucosal barrier. Paneth cells sense bacteria via MyD88-dependent toll-like receptor (TLR) activation, which then leads to degranulation and release of their AMPs. Intestinal derived-IL-22 promotes homeostasis and induces RegIII production. Intestinal dendritic cells help organize the gut microbiota by sampling luminal bacteria and presenting their antigens, thereby driving B-cells to become IgA-producing plasma cells.

The relationship between the host and its microbiota is symbiotic and mutually advantageous. The host offers a suitable environment and essential nutrients for microbial growth, while the gut microbiota contributes to the host’s health by supporting metabolic functions and promoting the maturation of the intestinal immune system [8]. Gut microbiota exerts its effects in the immune system through three main pathophysiological mechanisms: metabolic, structural, and protective. In the first mechanism, the gut microbiota ferments dietary fibers in the large intestine, leading to the production of metabolites that influence immunity, such as short-chain fatty acids (SCFA), including acetate, propionate, and butyrate. SCFAs enter host cells and interact with G-protein-coupled receptors (GPR41, GPR43 and GPR109A) located on epithelial and immune cells. In particular, butyrate engages GPR43 and promotes the induction of anti-inflammatory cytokines, such as IL-10 and TGF-β. Furthermore, SCFAs, especially butyrate, are the major and preferred source of energy for the colonic epithelium. Secondly, commensal microbial populations enhance tight junctions of the intestinal epithelium, creating a stable structural model, while some enterotoxins of pathogenic bacteria such as *E. coli*, *C. difficile* and *C. perfringens* increase intestinal permeability [9]. Tight junctions constitute an essential structural component that maintains epithelial barrier integrity by controlling trans-epithelial permeability. Thirdly, secretory IgA and antimicrobial peptides (AMP) secreted by Paneth cells in the intestinal mucosa, such as regenerating islet-derived III (RegIII) proteins, a family of secreted C-type lectin antimicrobial peptides, also play an important role in sustaining the integrity of the mucosal barrier. RegIII kills Gram-positive and some Gram-negative bacteria, maintaining intestinal homeostasis by creating a barrier between the gut microbiota and the host epithelium. In the absence of RegIII, bacteria colonize the epithelial surface more extensively, which in turn triggers stronger activation of adaptive immune responses. Additionally, epithelial cells and innate lymphoid cells (ILCs) cooperate to maintain intestinal homeostasis by providing antimicrobial defense and secreting cytokines. ILCs-derived IL-22 promotes epithelial repair and induces RegIII production [8]. Intestinal dendritic cells (DC) play a pivotal role in spatially organizing immune responses to gut microbiota by actively sampling luminal bacteria and presenting their antigens to the T-cells of the immune system. They induce B-cells to differentiate into IgA-producing plasma cells responsible for IgA secretion [10] (Figure 1).

## 4. Interaction Between Gut Microbiota and Cancer

Previous studies have confirmed that gut microbiota consists of more than 2000 species. It has been suggested that the onset and progression of several diseases may influence its composition [11]. Gut microbiota dysbiosis increases with age. Over the last decade, efforts to understand the possible association between the intestinal microbiome and carcinogenesis have become an increasing focus of research. Several studies have shown that gut microbiota influences the oncogenesis and progression of breast, pancreatic and hepatocellular carcinoma [12]. Inflammation is considered to represent the central link between microbiome and cancer. It is associated with an increase in disease-related pathogens and a decrease in protective bacteria, leading to microbial dysbiosis [13]. The disruption of the epithelial barrier induced by dysbiosis enables some gut microbiota to access local lymph nodes or reach the bloodstream. This is followed by the activation of the immune system and alterations of intracellular signaling pathways, factors that may have an impact on carcinogenesis [11].

As already mentioned, interactions between host and microbes may influence carcinogenesis through several pathways, including chronic inflammation and shifts in the microenvironment. These effects can arise from the microbial community as a whole or from specific microorganisms such as *Helicobacter pylori*, which has been linked to an increased risk of gastric adenocarcinoma and gastric mucosa-associated lymphoid tissue (MALT) lymphoma [14]. *H. pylori* activates pro-inflammatory cyclooxygenase (COX) enzymes, particularly COX-2, which is upregulated by cytokines such as TNF-α, IL-1β and IFN-γ. The resulting inflammation generates mutagenic molecules such as reactive nitrogen species from inducible nitric oxide synthase (iNOS) that can damage DNA and contribute to carcinogenesis [15]. This example illustrates how microbial activity can lead to carcinogenesis through inflammation-mediated mechanisms.

As far as leukemogenesis is concerned, a preclinical study aimed to investigate the relationship between host genetics and gut microbiota in a mouse model of acute lymphoblastic leukemia (ALL). The researchers showed that a subclinical PAX5 predisposition shapes the composition of gut microbiota. Their data indicate that leukemia development in genetically susceptible mice results from the loss of commensal microbial populations rather than the presence of specific bacterial species. In contrast, commensals such as *Alistipes* spp. where found to influence the tumor microenvironment by inducing tumor-associated myeloid cells to produce tumor necrosis factor-α (TNF-α) through TLR-4 signaling, thereby contributing to tumor eradication [16]. Moreover, research on the intestinal microbiome in ALL has revealed a characteristic dysbiotic profile, marked by a significantly increased abundance of specific bacterial species such as *Bacteroides clarus*, *Roseburia faecis* and *Fusobacterium naviforme* compared to age-matched healthy controls. Furthermore, the gut microbiota has been shown to exert a substantial influence on hematopoiesis, metabolism and immune regulation and may, potentially, contribute to the development of AML. The complex interplay between the human microbiota and AML pathogenesis is still not fully defined. Microbiota-driven alterations in metabolic pathways are thought to potentially promote leukemogenesis and support the survival of leukemic blasts [17]. In patients with AML, randomization-based studies have demonstrated a causal relationship between gut microbiota composition and leukemia risk, implicating *Blautia* and *Rikenellaceae* RC9 gut group as pro-leukemogenic taxa [18]. These findings suggest the potential contribution of microbial dysbiosis to the formation of a microenvironment that supports leukemic cells and the modulation of disease risk [19].

## 5. Gut Microbiota Regulates AML in a Metabolic-Dependent Manner

**Figure 2:**
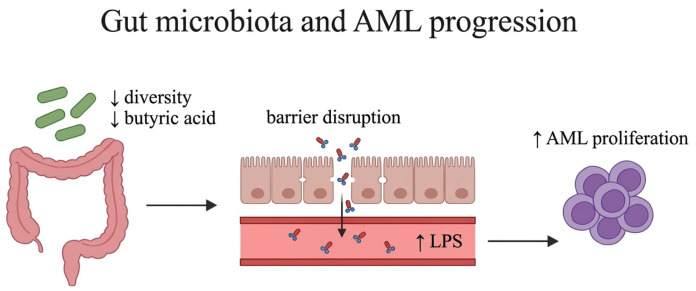
**The impact of gut microbiota on acute myeloid leukemia (AML) progression:** Patients with AML have decreased diversity in gut microbiota and significantly lower fecal butyric acid content than healthy individuals. Butyric acid deficiency could disrupt the intestinal barrier and enable bacterial lipopolysaccharide (LPS) release into the bloodstream. In vitro data have indicated that LPS enhances leukemia cell proliferation, indicating that low butyric acid and high LPS levels may promote AML progression.

Gut microbiota and SCFAs play a crucial role in the growth of intestinal epithelial cells and influence their differentiation and repair. Butyrate, an important SCFA, is derived from the metabolism of intestinal bacteria with butyrate-producing capacity, especially *Faecalibacterium* spp. Patients with AML demonstrate decreased diversity in gut microbiota and have significantly lower fecal butyric acid content as compared with healthy individuals. Butyric acid provides energy to intestinal cells and its deficiency could result in intestinal barrier disruption and impairment of mucosal immunity. In a clinical study involving treatment-naïve patients with AML, Wang and colleagues showed that decreased butyric acid and impaired intestinal barrier enable lipopolysaccharide (LPS) leakage into the blood circulation. Additionally, they found that LPS increases proliferation in leukemia cells, suggesting that the absence of butyric acid and high concentrations of LPS may have an impact on AML progression [20]. Figure 2 shows the impact of the intestinal microbiome on AML progression.

## 6. Is Altered Microbial Diversity Associated with Higher Risk of Infection in AML?

Infection due to chemotherapy-induced myelosuppression is a common complication and an important cause of death in patients with AML. Gut microbiota regulates immune system function and is associated with resistance to infections. Several studies have reported that intestinal microbiota can produce inhibitory products to suppress vancomycin-resistant enterococci. In patients with AML, the exposure to antibiotics and especially carbapenems during periods of neutropenia is common. It has been shown that carbapenems such as meropenem decrease gut microbiota diversity and increase colonization with opportunistic pathogens such as vancomycin-resistant enterococci [2].

In a clinical study, in which 97 patients with AML were enrolled, bacterial flora in fecal specimens was analyzed every two weeks from induction chemotherapy to neutrophil recovery. It was found that greater microbial diversity was associated with a lower risk of infection during induction chemotherapy. Additionally, it was reported that flora diversity gradually decreased after induction chemotherapy [2]. Other studies have reported that high pretreatment levels of *Proteobacteria* are associated with febrile neutropenia. Moreover, an increased abundance of *Enterococcaceae* or *Streptococcaceae* leads to a higher risk of later infection, and a higher temporal variability of intestinal microbiome is associated with an elevated infection risk at three months post-induction chemotherapy among patients with AML [21].

## 7. The Role of Antibiotics in Gut Dysbiosis in AML

In patients with AML, the administration of broad-spectrum antibiotics during the neutropenic period is usually unavoidable. Recent evidence indicates a strong correlation between antibiotics and gut dysbiosis. Previous studies have shown suppression of microbial diversity and overgrowth of *Enterococci* induced by antibiotics in patients with AML [22]. The genus *Enterococcus* consists of multi-drug resistant organisms (MDROs) and benefits from the depletion of Gram-negative bacteria induced by the use of antibiotics. In a healthy intestinal environment, Gram-negative bacteria induce the production of antimicrobial peptides, such as RegIIIγ, a crucial bactericidal C-type lectin produced by intestinal Paneth cells, that binds peptidoglycan to kill Gram-positive bacteria and protect the epithelial barrier [23]. The widespread use of antibiotics leads to the eradication of Gram-negative bacteria and, as a consequence, diminished RegIIIγ production and reduced defense against *Enterococci* [11]. Moreover, the intestinal overgrowth of bacteria is a predominant feature of AML patients developing neutropenic enterocolitis [24]. The proliferation of *Enterococcus* species can aggravate pathogenicity by altering gut metabolic pathways in ways that favor the growth of other pathogens. For instance, *Enterococcus* can promote *Clostridium difficile* virulence [25].

Antimicrobial resistance has become one of the most critical health issues and the major contributor to this crisis is the inappropriate use of antibiotics that disrupts the composition of the healthy intestinal microbiota [26]. It is well established that the gut microbiota plays a central role in colonization resistance [27]. Under normal conditions, a balanced microbiome suppresses colonization with pathogenic bacteria through competitive niche occupation, secretion of antimicrobial compounds and modulation of host immune responses. In contrast, antibiotic-induced dysbiosis disrupts these protective mechanisms and facilitates the proliferation of multi-drug-resistant organisms, especially those belonging to the ESKAPE group (*Enterococcus faecium*, *Staphylococcus aureus*, *Klebsiella pneumoniae*, *Pseudomonas aeruginosa*, *Acinetobacter baumannii* and *Enterobacter* spp.) [28]. In a comprehensive case–control clinical study enrolling three distinct cohorts (patients with MDROs infection, asymptomatic MDROs carriers, and healthy controls), a pronounced compositional shift in the intestinal microbiome was evident, marked by a transition from a “*Bacteroides*-dominant” to an “*Enterococcus*-dominant” enterotype in association with MDROs status. A particularly notable finding was the loss of beneficial commensal species such as *Faecalibacterium* in MDROs-positive patients, creating a gut ecosystem permissive to colonization with MDROs. *Faecalibacterium* has been recognized as a valuable biomarker of gut health and exerts direct antagonistic activity against *Klebsiella pneumoniae* [26]. Thus, shortening the duration of empirical broad-spectrum antibiotic treatment (EBAT) offers several advantages, including a reduced risk of colonization and subsequent infection with resistant bacteria. The How Long Trial showed that it is safe to discontinue EBAT in neutropenic patients once they have been afebrile and hemodynamically stable for 72 h, rather than continuing EBAT until neutrophil recovery [29].

## 8. The Effect of Chemotherapy Drugs on Gut Microbiota in AML

Several studies have demonstrated that gut microbiota is associated with both drug efficacy and drug side effects. It plays a critical role in drug metabolism during enterohepatic circulation either prior to drug absorption or through a variety of microbial enzymatic reactions. The gut microbiota exhibits a wide range of metabolic activities and can influence the pharmacokinetics of administered drugs as well as their therapeutic outcomes [30]. Antineoplastic drugs can directly act on the gut microbiota and destroy the epithelium and the intestinal barrier, resulting in imbalances in the composition and function of intestinal microbes and intestinal dysbiosis. Conversely, the gut microbiota can alter a response to a drug by enzymatically shifting its structure, which may influence its bioavailability, bioactivity and toxicity. As a result, dysbiosis not only induces local adverse events in the intestinal tract but can also alter the efficacy of chemotherapy, promote drug resistance and influence treatment outcome in acute leukemia [11]. Overall, alterations in gut microbiota composition have been linked to resistance to chemotherapeutic agents, whereas supplementation with specific bacterial species can restore responsiveness to anticancer treatments. Growing evidence also suggests that targeted modulation of the gut microbiome represents a promising approach to enhance the efficacy of anticancer agents [31].

In a metagenomic analysis aiming to understand the interaction between chemotherapy and gut microbiota, Luo et al. showed decreased microbial diversity two weeks after chemotherapy in patients with acute leukemia. In particular, they noted a decrease in certain beneficial bacteria belonging to the *Lachnospiraceae* family and an increase in pathogenic bacteria such as *Klebsiella* spp., *Streptococcus anginosus* and *Acinetobacter johnsonni*, suggesting intestinal dysbiosis. Reduction in the abundance of bacteria responsible for producing butyrate, a crucial component for intestinal integrity, may be responsible for the deregulation of the intestinal barrier [32].

Renga et al. demonstrated that CPX-351 (Vyxeos) may be beneficial for maintaining a functional mucosal barrier and preserving gut microbiota function as compared with “7 + 3” induction in patients with AML. Patients who received “7 + 3” in this clinical study had different microbial compositions and deregulated metabolic activity compared to the microbial eubiosis of the AML patients receiving CPX-351. They found that CPX-351 activated the host protective aryl hydrocarbon receptor (AhR) pathway, which promotes the epithelial barrier protection and strengthening. It has been reported that the liposome is likely responsible for this beneficial effect, since liposomes can influence the activity of immune cells and microbes [5]. There is no data regarding gut microbiota function in patients with AML treated with the combination of azacitidine with venetoclax.

## 9. The Involvement of Gut Microbiota in the Pathophysiology of Chemotherapy-Induced Gastrointestinal Mucositis

Gastrointestinal mucositis is a common side effect of antitumoral drugs manifested by nausea, vomiting, diarrhea and abdominal pain. The chemotherapy-induced loss of enterocytes results in epithelial injury and disrupts the epithelial barrier [33]. According to the model proposed by Sonis for the pathophysiology of GI mucositis, five phases are involved: (1) production of reactive oxygen species leading to oxidative stress and the activation of nuclear factor kappa B (NF-κB); (2) induction of TNF-a followed by inflammation; (3) intensification of the inflammation by production of additional inflammatory mediators resulting in tissue inflammation and apoptosis; (4) disruption of the epithelial barrier inducing bacterial translocation; and (5) cell proliferation that indicates the healing phase [34]. However, this five-phase model does not include the role of the intestinal microbiome in chemotherapy-induced gastrointestinal mucositis.

In recent years, studies have tried to show the potential impact of the intestinal microbiome on the development and progression of intestinal mucositis. Van Vliet et al. reported that several intestinal bacteria are capable of inhibiting NF-κB activation, playing a protective role against inflammation. In particular, *Bacteroides thetaiotaomicron* and *Bifidobacterium infantis* decrease NF-κB activation and IL-6 levels. Moreover, other intestinal bacteria produce SCFAs, which suppress inflammatory responses [34]. It has also been reported that the intestinal microbiome can influence intestinal permeability through proteins produced by bacteria. *Bifidobacteria* and *Lactobaccilli* are both capable of producing proteins forming tight junctions, decreasing intestinal permeability [34]. Ewaschuk et al. showed that the administration of *Bifidobacterium infantis* protects intestinal permeability and enhances tight junctions [35]. Additionally, some bacterial strains in the human intestinal microbiome can regulate genes encoding mucins and, thus, play a significant role in the composition of the mucus layer [36]. These mechanisms highlight the involvement of the intestinal microbiota in the maintenance of intestinal health and its potential influence in the pathophysiology of gastrointestinal mucositis.

## 10. The Role of Diet in Shaping Gut Microbiome

Patients with AML undergoing induction chemotherapy or after hematopoietic stem-cell transplantation (HSCT) usually receive a low-bacterial neutropenic diet to reduce the infection risk from ingested bacteria. Recent surveys indicate that more than 80% of bone marrow transplantation centers still implement a low-microbial protective diet during the neutropenic period after HSCT, despite the absence of standardized dietary prescription protocols [37]. In such patients, raw fruits and vegetables are considered to be a source of infection and neutropenic diets aim to minimize the bacterial load that could cause infectious complications [38]. Additionally, the presence of gastrointestinal mucositis with the ensuing translocation of Gram-negative bacteria from the intestinal lumen to lymphoid tissues and blood, during cytopenic phases, further exacerbates the possibility of infection [39].

Diet generally acts as a significant regulator in forming the structure and function of the intestinal microbiome. There is ongoing debate regarding the impact of a neutropenic diet on the gut microbiota, with growing concern that such dietary restrictions may negatively affect microbial diversity and intestinal health through the reduction in high-fiber-containing food [40]. Given that the gut microbiome is a key component of host defense, such diet-induced dysbiosis may increase susceptibility to bloodstream infections and promote the growth of antibiotic-resistant pathogens. Furthermore, dietary restrictions can reduce nutritional intake, diminish the enjoyment of eating and negatively affect quality of life during chemotherapy [41]. It is well known that after HSCT, weight loss and malnutrition can develop quickly and may have serious consequences. The inclusion of certain foods in patients’ nutrition, such as fresh fruits, has been associated with improvements in gastrointestinal symptoms, including nausea and vomiting [37]. Evidence from multiple studies indicates no harm in permitting patients to consume well-washed raw fruits, vegetables and pasteurized dairy products such as yogurt [41].

A 120-day longitudinal clinical study (30 days of sterile diet followed by 90 days of normal diet) in leukemia patients showed that sterile diet disrupts the microbial balance, impairs the production of SCFAs and results in the proliferation of potential pathogens. On the other hand, a normal diet promotes the abundance of beneficial bacteria and the recovery of gut microbiota, since it offers microbial taxa that participate in the process of restoration [40].

Results from a clinical study of 20 patients with leukemia showed no significant difference in the rates of gut colonization by Gram-negative bacteria in those who followed a restricted-neutropenic diet compared to a normal hospital diet [39]. In another randomized trial conducted on 153 patients with newly diagnosed AML, the use of a cooked diet was compared to a non-cooked diet containing fresh fruit and vegetables. It was found that rates of major infection and death were similar in the two arms [42]. These findings suggest that the routine use of a neutropenic diet needs to be re-evaluated and that a more liberal approach may be more appropriate, since it will also have a favorable effect on patients’ appetite and psychological well-being.

## 11. Gut Microbiota and Cancer Cachexia in AML Patients

Patients with AML and gastrointestinal mucositis following chemotherapy often develop diarrhea, abdominal pain and discomfort, which may lead to a reduced oral intake, impaired nutritional absorption and weight loss. This phenomenon may contribute to the development of cancer cachexia. Cancer cachexia is characterized by involuntary and progressive weight loss, with ongoing loss of skeletal muscle mass [43]. It has a significant negative impact on survival and it is reported that higher mortality occurs in malnourished and underweight patients undergoing allogenic hematopoietic stem cell transplantation (allo-HSCT) [44]. Cachexia remains an unmet medical need in the field of oncology due, in part, to limited evidence for effective therapeutic targets, since the biological mechanisms underlying this metabolic syndrome are not completely understood [43].

Recent research has focused on investigating the potential relationship between cancer cachexia and gut microbiota. Accumulating evidence from preclinical studies based on mouse models links metabolic aspects of cancer cachexia to gut microbiota [43]. The gut microbiota has been shown to influence dietary energy extraction, systemic inflammatory responses, intestinal barrier integrity and insulin sensitivity, key metabolic processes that are disrupted in cancer cachexia [45]. Cachexia is characterized by systemic inflammation mediated by cytokines (TNF-α, IL-1, IL-6), which contribute to enhanced skeletal muscle protein degradation and reduce appetite and food intake. This inflammatory process disrupts normal metabolic homeostasis, resulting in increased muscle wasting and impaired nutrient utilization [46]. Gut microbiota influences systemic inflammation via the intestinal barrier. The intestinal barrier, acting as a selective interface between the lumen and the intestinal environment, prevents bacterial translocation into circulation and inflammatory activation [43]. Additionally, several studies have reported associations between the gut microbiome and muscle mass. In mouse models of chronic intestinal inflammation, colonization with *Escherichia coli* was found to prevent skeletal muscle atrophy through activation of the insulin-like growth factor (IGF-1)/PI3K/AKT pathway [43]. Additionally, gut microbiota influences muscle metabolism by modulating amino acid availability through metabolite-mediated effects on glucose metabolism and muscle glycogen content. Reduced SCFA levels have been observed in cachectic cancer patients, with acetate showing a significant decrease. Acetate is known to contribute to body weight regulation, energy metabolism, and insulin sensitivity [45]. Further support for the involvement of gut microbiota in cancer cachexia comes from studies demonstrating beneficial outcomes of microbiota-targeting interventions [46]. Various approaches can be used to modulate the gut microbiota, including probiotics, prebiotics and synbiotics [43]. Collectively, these observations support further research aimed at elucidating the role of the intestinal microbiome in cancer cachexia and investigating its potential as a novel therapeutic target.

## 12. The Role of Intestinal Microbiome in Graft-Versus Host Disease

Graft-versus-host disease (GvHD) is the major complication and a main cause of morbidity and mortality in patients with AML after allo-HSCT. It is characterized by the attack of allogenic T-cells on healthy host tissues. Acute GvHD occurs within 100 days after transplantation; the most significant risk factor in its development is HLA mismatch. The incidence of acute GvHD is around 30–50% in HLA fully matched allo-HSCT [47]. The 2-year survival of patients with grade 3 and 4 GvHD is 25–30% and 1–2%, respectively [12]. Experimental data indicate that acute GvHD develops in three phases: the first phase involves epithelial cell injury caused by the conditioning regimen, resulting in the secretion of cytokines and the activation of host antigen-presenting cells. The second phase is characterized by activation of donor T lymphocytes by antigens presented by the recipient’s dendritic cells and production of Th1 cytokines, followed by the third phase in which the activated T-cells act direct to the tissues, causing cell death and tissue damage, especially to the skin, gut and liver [12]. The main cytokines involved in the pathophysiology of GvHD are TNF-α and IL-1. It is suggested that the initiation of GvHD occurs when alloreactive type 1 helper T-cells produce IFN-γ, which induces the secretion of TNF-α and IL-1 [48].

**Figure 3:**
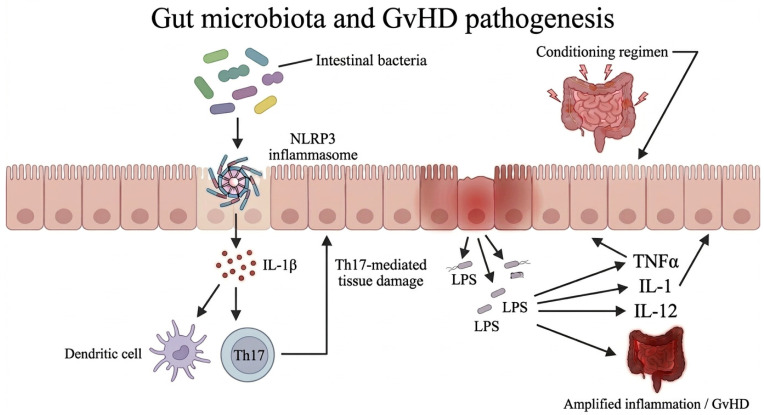
**Microbiota-driven cytokine storm in acute graft-versus-host disease (GvHD).** Intestinal bacteria have been shown to promote NLRP3 inflammasome-mediated secretion of IL-1β. IL-1β released from intestinal cells modulates dendritic cells and Th-17 cells, which in turn amplify epithelial injury. Moreover, injury of the intestinal mucosa induced by conditional regimens during the first phase of GvHD and by cytokines in the second phase results in the translocation of lipopolysaccharide (LPS) in blood. LPS contributes to GvHD pathogenesis by inducing inflammatory cytokines in monocytes-macrophages and T-cells, including TNF-α, IL-1, and IL-12, which further damage the intestinal epithelium and amplify inflammation, leading to cytokine storm.

Increasing evidence has shown that the gastrointestinal tract has a predominant role in GvHD pathogenesis and in its characteristic “cytokine storm”. Studies have demonstrated that intestinal bacteria are involved in the NLRP3 inflammasome-mediated secretion of IL-1β after conditioning. Il-1β is produced by intestinal cells and influences dendritic cells and Th17 cells [48]. Bacterial LPS, an essential component of the intestinal flora, is also involved in the pathogenesis of GvHD, because it acts as a stimulator of cytokine production, such as TNF-α, IL-1 and IL-12. Thus, LPS may offer a second signal to the secretion of TNFα, in addition to IFN-γ. Moreover, the sensitivity of monocytes and macrophages to endogenous LPS is intensified by the secretion of IFN-γ by alloreactive T-cells during GvHD [49] (Figure 3).

Injury of the intestinal mucosa induced by conditioning regimens during the first phase and by cytokines in the second phase results in the translocation of LPS in blood. Subsequently, LPS can intensify inflammation by stimulating more cytokines produced by gut lymphocytes and macrophages. As a result, tissue damage is intensified and it can amplify the inflammatory response, leading to tissue destruction and “cytokine storm” [49] (Figure 3). On the other hand, studies have revealed that IL-22 and IL-25 may have a protective role in the pathogenesis of GvHD. Intestinal stem cells (ISC), which are responsible for the regeneration and healing of intestinal epithelium after damage, express the IL-22 receptor. Intestinal IL-22 derives from innate lymphoid cells. It has been shown that IL-22 deficiency is associated with epithelium apoptosis, reduction of ISCs and loss of epithelial integrity [50]. Goblet cells are also critical for the structure of the mucus layer and intestinal integrity. Loss of goblet cells is an important histological finding in gastrointestinal GvHD [51]. It has been demonstrated that the administration of IL-25 promotes the expansion of goblet cells and reduces bacterial translocation, IFN-γ secretion and ameliorates GvHD [48].

## 13. Microbial Diversity in Acute GvHD

**Figure 4:**
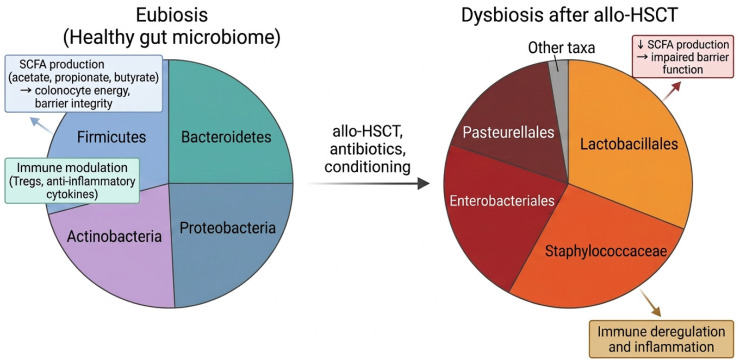
**Schematic representation of gut microbiome eubiosis (left) and dysbiosis associated with allo-HSCT (right).** The healthy gut microbiome is dominated by *Firmicutes*, *Bacteroidetes*, *Proteobacteria* and *Actinobacteria*, supporting SCFAs production and immune modulation. In contrast, dysbiosis after allo-HSCT is characterized by overpresentation of *Lactobacillales*, *Staphylococcaceae*, *Enterobacteriales* and *Pasteurellales* with reduced diversity, decreased SCFA production, impaired barrier function and immune deregulation. SCFA, short-chain fatty acids; allo-HCST, allogenic stem cell transplantation.

Alterations of intestinal microbial composition and reduced microbial diversity are observed in patients undergoing allo-HSCT and are more pronounced in acute GvHD. In a clinical study analyzing fecal samples from 1362 allo-HSCT recipients, it was found that lower diversity was associated with higher GvHD-related mortality [52]. Regarding the composition of gut microbiota after allo-HSCT, evidence has shown that *Lactobacillales*, *Staphylococcaceae*, *Enterobacteriales* and *Pasteurellales* are expanded, inducing microbial dysbiosis [51]. Another clinical study showed that alterations in gut microbiota during the early posttransplant period (1–28 days) are critical for the development of acute GvHD. It was also shown that a lower abundance of *Blautia* spp. and *Akkermansia muciniphila* was associated with GvHD development, severity and mortality [53]. Moreover, the composition of products derived from the intestinal microbiome (microbial metabolites) is also affected during microbial dysbiosis. Consequently, levels of SCFAs are decreased in patients with GvHD and one clinical study showed that the amount of butyrate was significantly diminished in the early post-transplant period in patients who developed gastrointestinal GvHD, rendering this metabolite a potential diagnostic marker [54] (Figure 4).

## 14. Therapeutic and Preventive Opportunities

### 14.1. Dietary Interventions Targeting Gut Microbiota: Probiotics, Prebiotics, Synbiotics

#### 14.1.1. Probiotics

Probiotics are beneficial microorganisms that, when administered in adequate amounts, provide health benefits to the host. In addition to restoring microbial balance, probiotic-derived metabolites can reduce the risk of intestinal infections and attenuate inflammation [12]. Probiotics exert antimicrobial and immunomodulatory effects, compete with pathogens for epithelial adhesion, enhance mucosal IgA production and exhibit anti-inflammatory properties [55]. During induction chemotherapy, antitumoral drugs disrupt the gut microbiota and damage the intestinal barrier, causing dysbiosis. Probiotics may help restore microbiota balance and protect the intestinal barrier, potentially supporting treatment outcomes and improving patients’ prognosis [56].

Among commonly used probiotics, *Lactobacillus* spp. and *Bifidobacterium* spp. are particularly prominent [57]. Specifically, *Bifidobacterium bifidum* has been applied in leukemia treatment to support immune homeostasis by enhancing IgA production, while lactic acid bacteria are capable of modulating epithelial tight junction proteins and, thereby enhancing intestinal barrier function [56]. Moreover, *Bifidobacterium* spp. suppress the production of inflammatory cytokines and also enhance the secretion of anti-inflammatory cytokines, including IL-10 [58]. Additionally, both *Lactobacillus* and *Bifidobacterium* have demonstrated potential anticancer properties in an in vitro study. *Lactobacillus* species have been shown to exhibit anticancer activity through modulation of immune responses and reduction of inflammation. Similarly, *Bifidobacterium* spp. can decrease cancer cell proliferation. The proposed mechanisms include inhibition of growth factor signaling pathways and activation of mitochondrial-mediated apoptosis pathways, which result in reduced proliferation of tumor cells [55]. Another probiotic with documented efficacy is *Saccharomyces boulardii*, a fungal microorganism classified as a yeast. Its probiotic effects arise from multiple mechanisms, including enhancement of gut barrier integrity, competitive exclusion of pathogens and modulation of the immune response [59]. Diarrhea and gastrointestinal colonization with potentially pathogenic bacteria, including *Clostridium difficile*, are major complications in the management of patients with AML. One potential strategy to mitigate these issues is the use of probiotics containing *Saccharomyces boulardii*, which may directly interact with and inhibit *Clostridium difficile* toxin A [60].

In summary, probiotics help maintain immune homeostasis within the gut microbiota and may mitigate certain complications associated with leukemia. They contribute to stabilizing the intestinal microenvironment and support host defense mechanisms. As previously described, a common complication of leukemia is bloodstream infection, which often arises from disruption of the intestinal barrier and dysbiosis of the gut microbiota. Supplementation of short-chain fatty acids has been shown to ameliorate the impact of bloodstream infections. These findings suggest that appropriate probiotic supplementation may improve intestinal health and thereby reduce the risk of leukemia-associated bloodstream infections [56].

Unlike other drug components, probiotics are administered as live microorganisms, which introduces a potential risk of infection. Although they are generally regarded as safe, adverse effects associated with probiotic use have been documented [61]. Infections caused by probiotics have been reported in individuals, including cases of fungemia due to *Saccharomyces* species [62]. Patients undergoing “7 + 3” chemotherapy typically receive antifungal prophylaxis, which may counteract the beneficial effects of probiotics based on *Saccharomyces* species. Moreover, immunosuppression has been identified as a predisposing factor for *Lactobacillus* bacteremia and such infections have also been described in patients with AML [63]. Consequently, a definitive causal relationship between probiotic use and a higher risk of infection in immunocompromised and hospitalized patients remains to be validated [64].

#### 14.1.2. Prebiotics

An alternative approach to modulate the intestinal microbiome involves the use of prebiotic fibers. These non-digestible compounds are fermented by intestinal bacteria, resulting in the production of SCFAs. Through this process, prebiotics can beneficially alter the composition of gut microbiota by selectively stimulating the growth of advantageous bacterial populations [43]. Prebiotics appear to increase the relative abundance of bifidobacteria within the gut microbiota. Compounds such as fructooligosaccharides (FOS) and galactooligosaccharides (GOS) fall into this category, exerting their effects primarily by enriching *Lactobacillus* and *Bifidobacterium* species [65].

#### 14.1.3. Symbiotics

Stimulation of probiotic bacteria by prebiotics modulates intestinal metabolic activity, helping to preserve intestinal structure and support the expansion of beneficial bacteria [66]. Synbiotics combine both probiotic and prebiotic components and were developed to address limitations in probiotic survival in the gastrointestinal tract. In fact, an optimized pairing of these two elements in a single formulation offers greater benefits than either a probiotic or a prebiotic used alone [67]. A combination of *Bifidobacterium* or *Lactobacillus* species with fructo-oligosaccharides is the most commonly used symbiotic formulation. The health benefits of symbiotic depend on the specific pairing of probiotic and prebiotic components [66]. Given that the term symbiotic implies synergy, it should ideally be reserved for products in which the prebiotic selectively promotes the growth or activity of the co-administered probiotic [67].

The gut microbiota undergoes significant alterations during both the development and treatment of AML and these shifts may influence the effectiveness of chemotherapy as well as overall prognosis. Evidence suggests that restoring microbial balance through probiotic, prebiotic and symbiotic supplementation could potentially contribute to improved clinical outcomes [56].

## 15. Fecal Microbiota Transplantation (FMT)

As previously mentioned, intensive chemotherapy for AML in combination with broad-spectrum antibiotics disrupt the normal gut microbiota balance, contributing to pathological conditions and a higher incidence of complications. In particular, chemotherapeutic drugs result in a substantial decline in microbial diversity and trigger microbial dysbiosis, marked by pronounced shifts in microbial communities and the predominance of pro-inflammatory bacterial species [68]. FMT has gained recognition as a promising therapeutic approach aimed at reshaping the gut microbiota to enhance host health. FMT involves administering processed stool from a healthy donor to a recipient with the purpose of reestablishing a stable and functionally balanced gut microbiome [46]. A growing body of evidence suggests that the intestinal microbiome plays a crucial role in acute GvHD following allo-HSCT. In this context, FMT may serve as a potential alternative for the management of aGvHD [69].

In a phase II single-arm multicenter clinical study assessing autologous fecal microbiota transfer in 25 patients with AML who underwent intensive chemotherapy combined with antibiotic therapy, it was observed that autologous FMT successfully restored the microbiota after its disruption, as demonstrated by microbial diversity indices returning to baseline levels after treatment. FMT led to the reestablishment of *Clostridiales*, *Lachnospiraceae* and *Ruminococcaceae* families to levels close to the original pre-chemotherapy abundance, while pro-inflammatory bacteria such as *Enterobacteriaceae* and *Enterococcaceae* were reduced to low levels [68]. This is important because the overgrowth of *enterococci* has been linked to the development of aGvHD, bacteremia and higher mortality [70]. Consequently, these findings indicate that autologous FMT enables AML patients to proceed to allo-HSCT with a more resilient and protective gut microbiota [68]. Furthermore, FMT has proven effective in correcting dysbiosis and managing cases of refractory acute GvHD. Evidence has shown that FMT proves beneficial not only in reducing fecal abundance of enterococci, but also in restoring *Collinsella*, whose depletion has been linked to febrile neutropenia after allo-HSCT [70]. Regarding chronic GvHD (cGvHD), a single-center clinical study was conducted to assess the effectiveness and safety of FMT. It was observed that patients with cGvHD were also found to have gut microbiota dysbiosis, characterized by the overgrowth of *Enterobacteriaceae* and that FMT decreased the levels of these pathogenic bacteria while promoting the growth of SCFA-producing bacteria, such as *Ruminococcaceae* [71]. In summary, FMT is effective in ameliorating post-chemotherapy and GvHD-associated dysbiosis by restoring the gut microbiota and reducing the abundance of pathogenic bacteria that contribute to infectious complications.

However, there are several aspects of FMT that remain insufficiently understood and require further clarification, including the long-term safety profile and optimal donor–recipient screening strategies [72]. Clarifying these aspects will be essential for the safe incorporation of FMT into oncological practice and for ultimately enhancing patient outcomes [73]. Most FMT-related adverse events are mild and include symptoms such as diarrhea, abdominal discomfort and flatulence, which generally subside over the course of follow-up [72]. Nevertheless, despite donor screening procedures, a residual risk of transmitting pathogens through FMT persists. Bacterial, fungal, viral or parasitic agents may be conveyed to the recipient and can pose a substantial danger to immunocompromised individuals. In this context, episodes of *E. coli* bacteremia and recurrence of *C. difficile* colitis have been documented after FMT in immunocompromised patients [73]. Thus, donor selection in oncology remains highly challenging. A central difficulty is the identification of asymptomatic carriers of pathogens such as *Enterobacteriaceae*, *C. difficile* and enteric viruses. Current evidence indicates that only 10–15% of potential donors meet eligibility criteria after comprehensive evaluation, including medical history, serological testing, stool pathogen screening and assessment of antibiotic resistance profile [74]. Undoubtedly, FMT appears promising in restoring microbial balance, attenuating GvHD severity and potentially improving survival. Nonetheless, despite these encouraging preliminary data, further work is required to develop and validate safe, standardized and effective treatment protocols [75].

## 16. Discussion

The gut microbiota plays a key role in health and disease. Its composition can be influenced by disease and research over the past decade has linked specific microbial patterns to cancer development and progression. Microbiota-driven inflammation disrupts the epithelial barrier, promotes the overgrowth of pathogenic bacteria, reduces protective species and activates immune and intracellular signaling pathways, all of which may contribute to carcinogenesis. Short-chain fatty acids, especially butyrate, produced by bacteria are essential for intestinal epithelial growth, differentiation and repair and also exert immunomodulatory and anti-inflammatory effects. In AML patients, gut microbiota diversity and fecal butyrate are reduced, leading to impaired intestinal barrier function and mucosal immunity, facilitating microbial translocation and systemic inflammation. Decreased butyrate allows LPS leakage into the blood, which can promote leukemia cell proliferation and contribute to AML progression. Furthermore, in patients with AML, broad-spectrum antibiotics used during neutropenia frequently induce gut dysbiosis, characterized by reduced microbial diversity and *Enterococcus* overgrowth, which has been linked to increased risk of bloodstream infections and adverse outcomes. The gut microbiota also affects drug metabolism, efficacy and toxicity. Chemotherapy-induced dysbiosis can decrease drug effectiveness and promote resistance, while restoring or modulating specific bacteria may improve response to treatment and reduce treatment-related toxicities. These clinical and treatment-related factors act as major confounders in microbiome studies, as neutropenia, prolonged hospitalization, recurrent infections and the widespread use of antibiotics can substantially reshape microbial communities. Their combined impact often obscures the distinction between alterations primarily driven by AML itself and those resulting from supportive care and anticancer treatment. Moreover, patients with AML undergoing allo-HSCT exhibit significant gut microbiota alterations and reduced microbial diversity, particularly those who develop acute GvHD. Evidence has shown that lower microbial diversity at the time of transplantation is associated with higher GvHD-related mortality, highlighting the prognostic importance of the intestinal microbiome. In this context, therapeutic approaches such as probiotics, prebiotics, symbiotics and FMT have emerged as promising strategies to help restore microbial balance and support homeostasis. Probiotics preserve immune balance in gut microbiota and may contribute to reducing specific complications related to leukemia and its treatment, while prebiotics and symbiotics can beneficially alter the composition of gut microbiota by selectively stimulating the growth of advantageous bacterial populations. Additionally, FMT has been shown to improve chemotherapy-induced and GvHD-related dysbiosis by restoring gut microbiota and decreasing the levels of pathogenic bacteria. Another important factor that significantly influences the gut microbiota is diet. Evidence suggests that a restrictive neutropenic diet disrupts microbial balance, whereas a more liberal, normal diet supports microbial diversity and promotes restoration of a healthier microbial community. Therefore, the routine use of a neutropenic diet should be reconsidered and nutritional strategies in patients with AML should be aligned with the goal of maintaining eubiosis whenever possible. Overall, microbiome-targeted interventions in AML, including dietary modulation, probiotic supplementation and FMT, appear promising for mitigating dysbiosis, enhancing treatment tolerance and potentially improving outcomes. However, current evidence is limited and, thus, well-designed clinical trials are needed to clarify their efficacy and safety in patients with AML. Before such approaches can be integrated into standard care, we need to find ways to define optimal patient selection and to establish standardized protocols. In summary, the gut microbiota in AML remains an area that requires further investigation. It undergoes crucial alterations throughout the course of the disease, influenced both by the leukemia itself and by therapeutic interventions and nutritional practices. Most available studies are observational and can demonstrate associations between specific microbiome features and outcomes rather than causality. Increasing evidence suggests that the intestinal microbiome is associated with disease-related complications and may play a role in their pathogenesis but also represents a modifiable target. Therefore, further research is essential to gain a deeper understanding of its role in AML with the aim of developing microbiome-based targeted strategies that could improve prognosis and enhance patients’ survival.

## 17. Conclusions

The gut microbiome is severely affected in patients with AML: it is altered by the disease itself and by therapeutic interventions used for its treatment. It plays a predominant role in the development of complications, including infections and treatment-related toxicities. Accumulating evidence indicates that dysbiosis and overgrowth of specific pathogens are associated with major complications, including bacteremia, chemotherapy-related mucositis and treatment resistance. AML-directed treatment often induces long-lasting alterations in the microbial community, barrier function and metabolite profile, which in turn further influence disease biology and therapeutic responsiveness. Consequently, targeting the intestinal microbiome through microbiome-directed therapies could enhance microbial diversity, mitigate complications and improve overall outcomes in patients with AML.

## 18. Future Directions

Having recognized the significance of the gut microbiota in AML, its restoration after chemotherapy treatment and prior to allo-HSCT appears to be an important therapeutic objective. Restoring a more balanced and diverse microbial ecosystem may contribute to improved mucosal barrier integrity, modulation of inflammation and enhanced immunity. In allo-HSCT, the gut microbiota is increasingly considered not only as a marker of increased risk of complications, but also a modifiable factor that can be reshaped to minimize post-transplant complications. FMT has emerged as a promising strategy to achieve this goal, as it promotes the recovery of microbial diversity and helps ameliorate dysbiosis caused by chemotherapy and antibiotics. In turn, this could allow patients to undergo allo-HSCT with a more stable intestinal microenvironment, potentially minimizing the risk of major complications, including gut GvHD.

## Data Availability

This is a review article with no new data created.

## Abbreviations

The following abbreviations are used in this manuscript:

AML	Acute myeloid leukemia
SCFA	Short-chain fatty acid
LPS	Lipopolysaccharide
ISC	Intestinal stem cells
FMT	Fecal microbiota transplantation
GvHD	Graft-versus-host disease
aGvHD	Acute graft-versus-host disease
cGvHD	Chronic graft-versus-host disease
MDROs	Multi-drug resistant organisms
